# Response to interpreting cross-sectional comparisons of menopausal status and brain outcomes

**DOI:** 10.1017/S0033291726104747

**Published:** 2026-07-13

**Authors:** Katharina Zühlsdorff, Christelle Langley, Richard Bethlehem, Varun Warrier, Rafael Romero Garcia, Barbara J. Sahakian

**Affiliations:** 1 Department of Psychology, University of Cambridge, Cambridge, UK; 2 Department of Psychiatry, University of Cambridge, Cambridge, UK; 3 Instituto de Biomedicina de Sevilla (IBiS) HUVR/CSIC/CIBERSAM, ISCIII, Department of Medical Physiology and Biophysics, University of Seville, Seville, Spain

Dear Editor-in-Chief,

We thank Catriona Keye (2026) for acknowledging the fact that this is the first study to investigate the effects of menopause and hormone replacement therapy (HRT) on mental health, cognition, and brain structure in a very large sample of women (*n* = 124,780 and *n* = 10,873 for neuroimaging) (Zühlsdorff et al., 2026). We were able to conduct this study because of the high-quality, extensive measures, including mental health and neuroimaging, in a large population of women in the UK Biobank, which provides a unique and very valuable resource of data.

We did not state in our article that the study findings were causal, but that they were associations.

In addition, in our article, we did note as a limitation that our design was cross-sectional and not longitudinal. We hope to conduct a longitudinal study when data for similarly large numbers of women are available to do so. However, the UK Biobank data start at age 40 years, and neuroimaging was not conducted at baseline.

We fully agree with Catriona Keye that further research is needed to determine all the factors influencing menopause and women’s mental health, and indeed we point to some of these factors, such as genetic predispositions and comorbidities, in the discussion section of our article.

Yours sincerely,

Katharina Zühlsdorff, Christelle Langley, Richard Bethlehem, Varun Warrier, Rafael Romero Garcia, and Barbara J. Sahakian
